# Alternative cryoprotective agent for corneal stroma-derived mesenchymal stromal cells for clinical applications

**DOI:** 10.1038/s41598-024-65469-4

**Published:** 2024-07-09

**Authors:** Kristoffer Larsen, Goran Petrovski, Gerard Boix-Lemonche

**Affiliations:** 1 Center for Eye Research and Innovative Diagnostics, Department of Ophthalmology, Institute of Clinical Medicine, Faculty of Medicine, University of Oslo, Oslo, Norway; 2 Department of Ophthalmology, Oslo University Hospital, Oslo, Norway; 3 School of Medicine, University of Split, 21000 Split, Croatia; 4 UKLONetwork, University St. Kliment Ohridski –Bitola, 7000 Bitola, North Macedonia

**Keywords:** Biochemistry, Biological techniques, Cell biology, Chemical biology, Stem cells

## Abstract

Cryopreservation of human corneal stroma-derived mesenchymal stromal cells (hCS-MSCs) with dimethylsulfoxide (DMSO) as a cryoprotective agent (CPA) has not been previously compared to that with glycerol under standard conditions. The hCS-MSCs were hereby cryopreserved with both compounds using a freezing rate of 1 °C/minute. The CPAs were tested by different concentrations in complete Minimum Essential Medium (MEM) approved for good manufacturing practice, and a medium frequently used in cell laboratory culturing—Dulbecco’s modified eagle serum. The hCS-MSCs were isolated from cadaveric human corneas obtained from the Norwegian Eye Bank, and immunophenotypically characterized by flow cytometry before and after cryopreservation. The survival rate, the cellular adhesion, proliferation and cell surface coverage after cryopreservation of hCS-MSCs has been studied. The hCS-MSCs were immunofluorescent stained and examined for their morphology microscopically. The results showed that cryopreservation of hCS-MSCs in MEM with 10% glycerol gives a higher proliferation rate compared to other cryopreserving media tested. Based on the results, hCS-MSCs can safely be cryopreserved using glycerol instead of the traditional use of DMSO.

## Introduction

In developing countries in Asia and Africa, corneal diseases requiring transplantation often stem from infectious and nutritional issues, while developed countries face inherited degenerative corneal disorders like keratoconus and Fuchs` corneal endothelial dystrophy that may require corneal transplantation or keratoplasty^1,2^, which is limited by donor scarcity and pre- and post- operative complications^2^. Taking this into consideration and the millions of patients waiting to be treated for corneal diseases, there is a need for alternatives using regenerative medicine technologies^3–5^.

Researchers are exploring cell-based therapy in different medical fields, including ocular diseases^3–5^. To succeed with such therapy, there are certain required traits for the specific cell lines^6^. Mesenchymal stromal/stem cells (MSCs) have been shown to possess several unique properties (e.g. multilineage differentiation potential, induction of tissue repairment, immunomodulation) for cell-based therapy^6^. Since 2005, some authors including our group have studied the multipotency of human corneal stroma-derived MSCs (hCS-MSCs)^7–11^. These cells are abundant in the central part of the corneal stroma and possess ability to reduce corneal scarring in a mouse model^7,10,11^, as well as wound healing properties in vitro^7^.

Challenges such as the lack of suitable preservation conditions in good manufacturing practices (GMP) persist^12,13^. Many authors employed basal growth media containing multiple nutrients, growth factors, and fetal bovine serum (FBS), which has been the gold standard medium supplement in many laboratories throughout the world^12^. However, the FBS can potentially represent a source of zoonotic infections and xenogenic antigens, both of which can impair the cell culture well-being^12^. The regulatory authorities strongly advise against using FBS or other animal derivatives for cell expansion, particularly for clinical GMP production^13,14^. Human platelet lysate (HPL), which contains a plethora of growth-promoting factors, is an excellent alternative to FBS for clinical MSC growth and development^12,15–17^.

Cryopreservation of cells is a flexible option which increases time-span of their use in research and medical treatment by many users^18,19^. However, there are challenges related to cryopreservation like ice crystal formation and changes in cell osmosis^14–16^, which can affect the cell survival rate^19,20^. Cryoprotective agents (CPAs) that diffuse into the cell before cryopreservation hinder crystal formation, which have advanced since they were first researched in the late 1800s^20,21^. The method of cryopreserving cells is optimized according to the cell type used^20^. Some cells are better cryopreserved using vitrification, a methodology that rapidly decreases the temperature of the cells allowing the liquids inside to reach a very high viscosity, preventing ice crystal formation^22^. Other cell types are better cryopreserved using a slower cooling rate and various CPAs^20^. For example, hematopoietic stem cells are traditionally frozen using slow cooling methods, while cells like human embryonic stem cells are better frozen using vitrification^23^.

CPAs like glycerol, dimethylsulfoxide (DMSO), ethylene glycol and propanediol (propylene glycol) are commonly used today. These molecules possess high water- solubility, small size molecules and preferentially low toxicity to the cells. DMSO, first synthesized in 1866, though popular, is toxic, raising concerns for GMP compliance^21^. It causes DNA methylation and changes to histones in the cell core^24^.

Glycerol was first used to preserve cells in 1949, and it is an abundant, cheap, and non-toxic chemical that does not require washing the cells after thawing from long-term cryopreservation, albeit higher viscosity^25^. As such, it could be a good candidate as a GMP cryoprotective agent.

This study aims to find the suitability of glycerol as a substitute for DMSO in cryopreserving hCS-MSCs in a potentially GMP-friendly manner. Various media types and glycerol concentrations are explored to assess their impact on maintaining or improving cell characteristics during cryopreservation compared to DMSO.

## Results

### Phenotype characterization of hCS-MSCs

**Figure 1:**
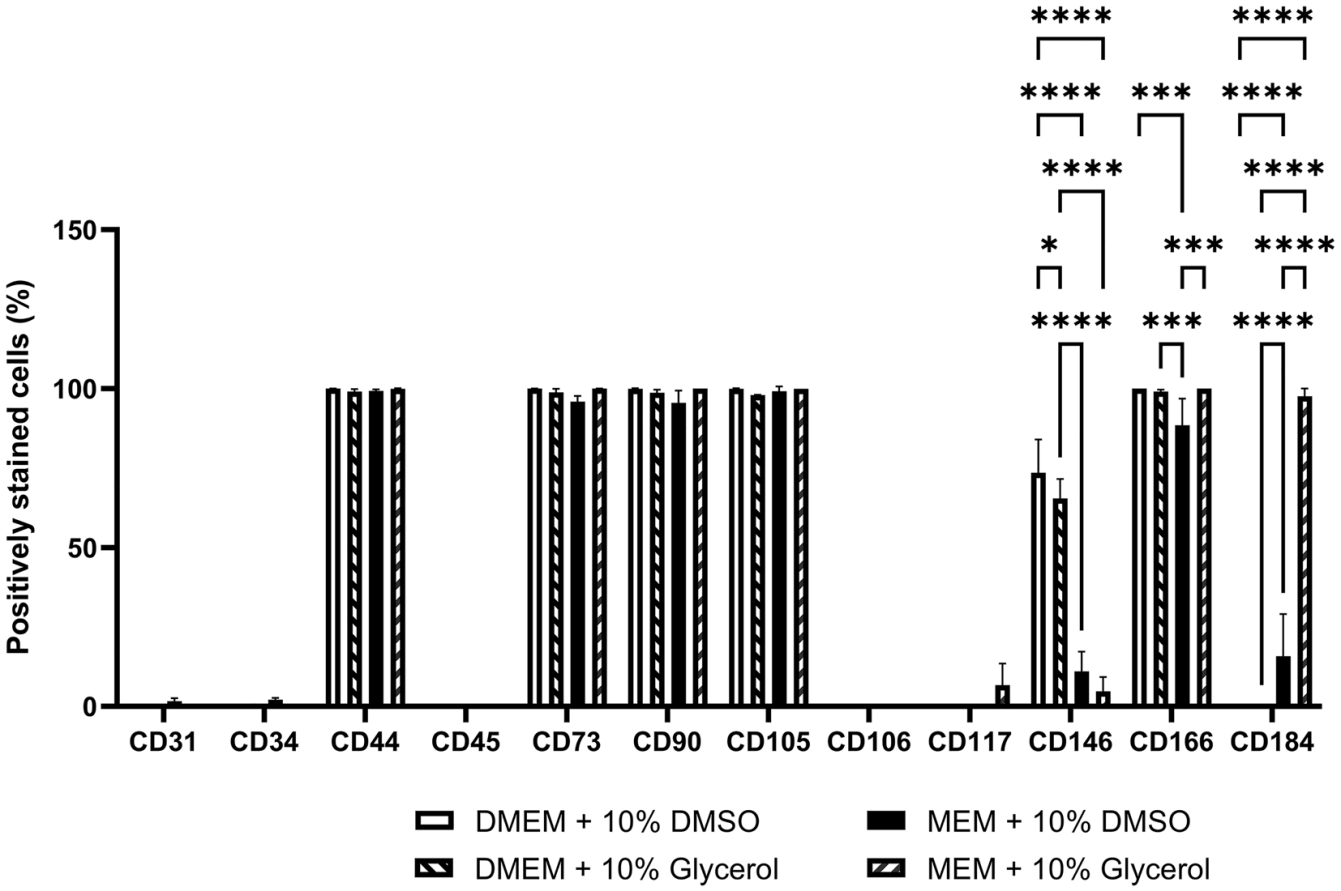
Comparison of surface protein expression between the use of selected CPAs in complete DMEM and MEM (GMP media) (*p < 0.05, ***p < 0.001, ****p < 0.0001). Data obtained with FACS analysis as shown in Supplementary Fig. 1.

The immunophenotype of the cryopreserved hCS-MSCs in 10% DMSO in complete DMEM showed cell surface marker presence of less than 5% for CD31/PECAM-1 (0.10 ± 0.00%), CD34 (0.13 ± 0.06%), CD45/PTPRC (0.03 ± 0.06%), and CD117/c-KIT (0.07 ± 0.06%). Over 95% of the cells expressed CD44/HCAM (99.97 ± 0.06%), CD73/NT5E (99.97 ± 0.06%), CD90/Thy-1 (99.93 ± 0.12%), CD105/Eng (99.90 ± 0.17%) and CD166/ALCAM (100.00 ± 0.00%), while other surfaces markers showed variable expression: CD106/VCAM-1 (0.33 ± 0.21%), CD146/MCAM (73.57 ± 10.39%) and CD184/CXCR4 (0.33 ± 0.21%) (See Supplementary Fig. S1 and Fig. 1).

The phenotype characterization of the hCS-MSCs frozen in 10% glycerol in complete DMEM presented less than 5% expression of CD31/PECAM-1 (0.30 ± 0.17%), CD34 (0.47 ± 0.012%), CD45/PTPRC (0.07 ± 0.06%), CD106/MCAV-1 (0.10 ± 0.00%), CD117/c-KIT (0.03 ± 0.06%) and CD184/CXCR4 (0.03 ± 0.06%). More than 95% of the cells expressed CD44/HCAM (99.07 ± 0.78%), CD73/NT5E (98.77 ± 1.10%), CD90/Thy-1 (98.67 ± 0.97%), CD105/Eng (97.90 ± 0.10%) and CD166/ALCAM (99.07 ± 0.5%). Marker CD146/MCAM (65.50 ± 6.12%) showed varied expression among the hCS-MSCs (See Supplementary Fig. S1 and Fig. 1).

The characterization of the hCS-MSCs cryopreserved in 10% DMSO in complete MEM presented less than 5% expression of CD31/PECAM-1 (1.67 ± 0.90%), CD34 (2.13 ± 0.59%), CD45/PTPRC (0.00 ± 0.00%), CD106/MCAV-1 (0.00 ± 0.00%) and CD117/c-KIT (0.00 ± 0.00%). More than 95% of the cells expressed CD44/HCAM (99.23 ± 0.47%), CD73/NT5E (95.87 ± 1.75%), CD90/Thy-1 (95.43 ± 3.85%) and CD105/Eng (99.10 ± 1.56%). Other surfaces markers showed varied expression among the hCS-MSCs, such as, CD146/MCAM (11.07 ± 6.14%), CD166/ALCAM (88.47 ± 8.34%) and CD184/CXCR4 (15.83 ± 13.27%) (See Supplementary Fig. S1 and Fig. 1).

The surface protein immunophenotype of the hCS-MSCs frozen in 10% glycerol in complete MEM presented less than 5% expression of CD31/PECAM-1 (0.00 ± 0.00%), CD34 (0.00 ± 0.00%) and CD45/PTPRC (0.00 ± 0.00%). More than 95% of the cells expressed CD44/HCAM (99.90 ± 0.17%), CD73/NT5E (99.97 ± 0.06%), CD90/Thy-1 (100.00 ± 0.00%), CD105/Eng (99.90 ± 0.00%) and CD166/ALCAM (100.00 ± 0.00%). Other surfaces markers showed varied expression among the hCS-MSCs, such as CD106/MCAV-1 (0.10 ± 0.00%), CD117/c-KIT (6.73 ± 6.70%) CD146/MCAM (4.77 ± 4.44%) and CD184/CXCR4 (97.53 ± 2.47%) (See Supplementary Fig. S1 and Fig. 1).

The hCS-MSCs cryopreserved with complete MEM media presented significant decrease in CD146- and an increase in CD184- expression compared to the hCS-MSCs frozen in complete DMEM media, with the hCS-MSCs preserved in 10% glycerol in MEM showing the most decrease in CD146 and the highest recovery of CD184 markers. The hCS-MSCs cryopreserved with 10% DMSO in complete MEM media presented significantly lower CD166 expression than the rest of the CPAs.

### Cell survival

**Figure 2:**
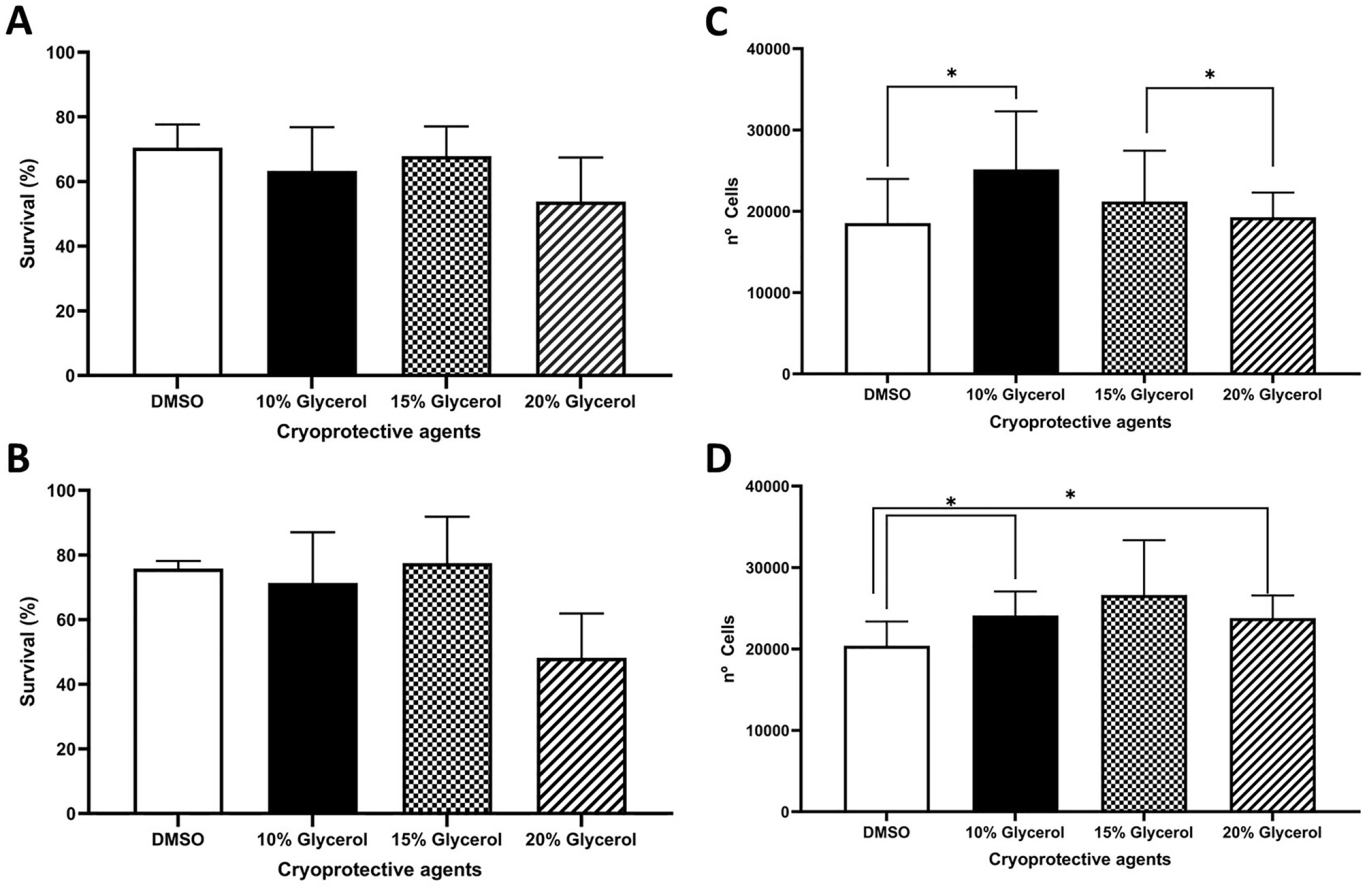
Survival studies and cell adhesion of hCS-MSCs under different conditions. (**A**,**B**) hCS-MSCs survival percentage in (**A**) complete DMEM and (**B**) complete MEM media after cryopreservation with different cryoprotective agents (10% DMSO, 10, 15 and 20% glycerol). (**C**,**D)** Cell adhesion to plastic surface after 6 h of incubation post-thawing (**C**) in complete DMEM and (**D**) complete MEM media (GMP media). All data expressed as average ± SD of 3 primary hCS-MSCs lines, (*p < 0.05).

The hCS-MSCs cryopreserved with different CPAs (10% DMSO, as well as 10, 15 and 20% Glycerol) in complete DMEM or MEM media showed similar survival rates after thawing the cells (approximately 70 ± 10%), without any statistically significant differences (Fig. 2A,B).

### Cell adhesion

The adhesion of hCS-MSCs after thawing them showed certain variability between the different CPAs. The hCS-MSCs cryopreserved with 10% glycerol in complete DMEM adhered to the plastic culture plate with a significantly higher number of cells in comparison to the cells cryopreserved with DMSO (Fig. 2C). Additionally, the cells cryopreserved with 15% glycerol adhered with a significantly higher number than the cells cryopreserved with 20% glycerol (Fig. 2C).

Similar findings could be seen with the hCS-MSCs cryopreserved in complete MEM (Fig. 2D). The cells cryopreserved with 10% or 20% glycerol showed a significantly higher adhesion compared to the cells cryopreserved with 10% DMSO (Fig. 2D).

### Cell proliferation

**Figure 3:**
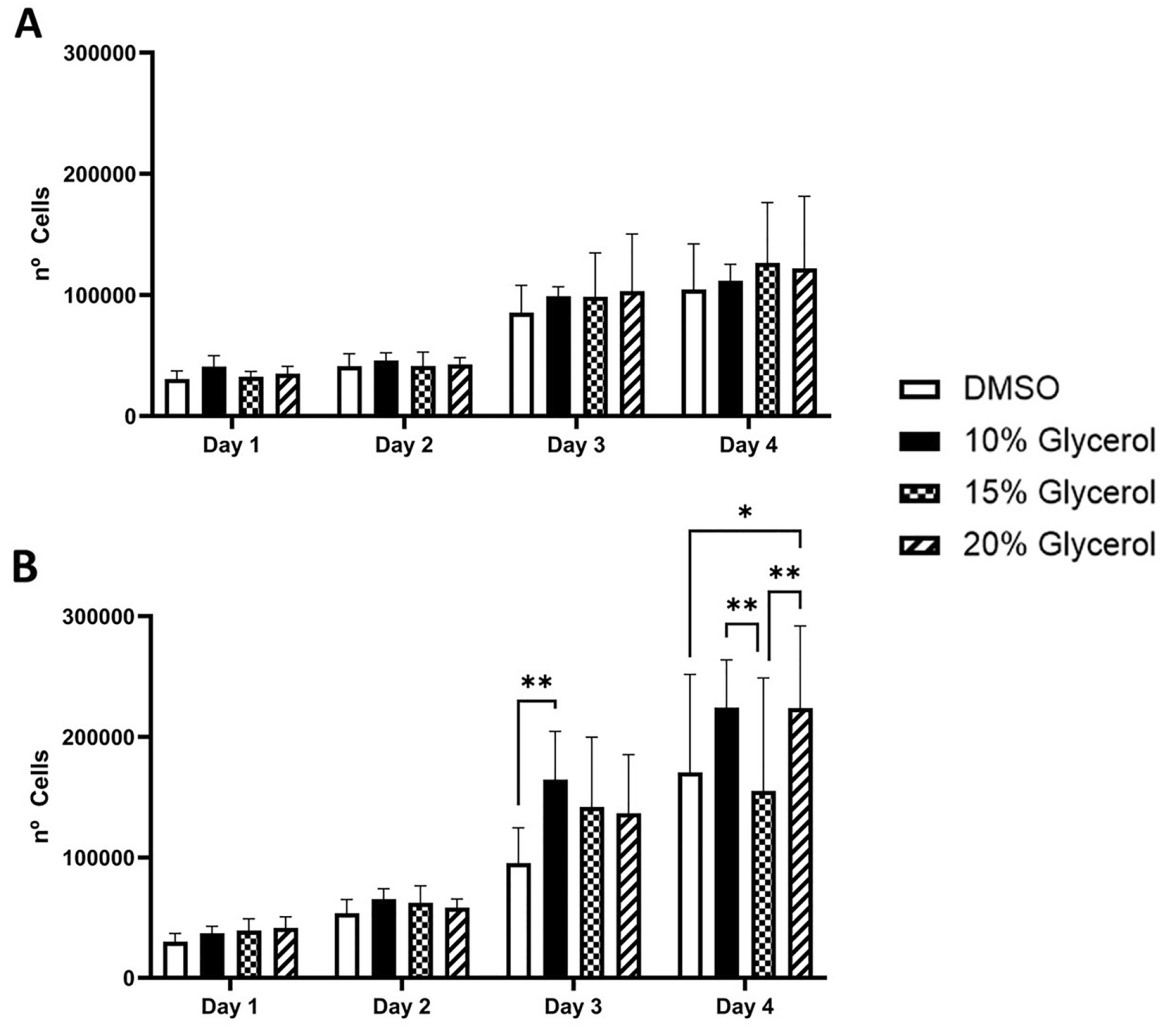
Human CS-MSCs proliferation after being cryopreserved with different CPAs (10% DMSO, 10%, 15% and 20% Glycerol). (**A**) complete DMEM media, (**B**) complete MEM media (GMP media). Data expressed as average ± SD of 3 primary hCS-MSCs lines (*p < 0.05, **p < 0.01).

The hCS-MSCs cryopreserved with any CPA in complete DMEM have not shown any significant difference in proliferation at any time point (Fig. 3A). Additionally, it was not observed any significant difference with the proliferation of the hCS-MSCs cryopreserved with any CPA in complete MEM during Day 1 and 2 (Fig. 3B). However, the hCS-MSCs cryopreserved with 10% glycerol in complete MEM at Day 3 showed a significantly higher proliferation in comparison to the cells preserved with 10% DMSO, but there was no significant difference between the other conditions (Fig. 3B). At Day 4, the hCS-MSCs cryopreserved with 10% glycerol in complete MEM showed a significantly higher proliferation in comparison to the cells preserved with 15% glycerol, while the hCS-MSCs cryopreserved with 20% glycerol presented a significantly higher proliferation in comparison to the cells cryopreserved with 15% glycerol and 10% DMSO in complete MEM (Fig. 3B).

### Cell surface covering, cell spreading and image analysis

**Figure 4:**
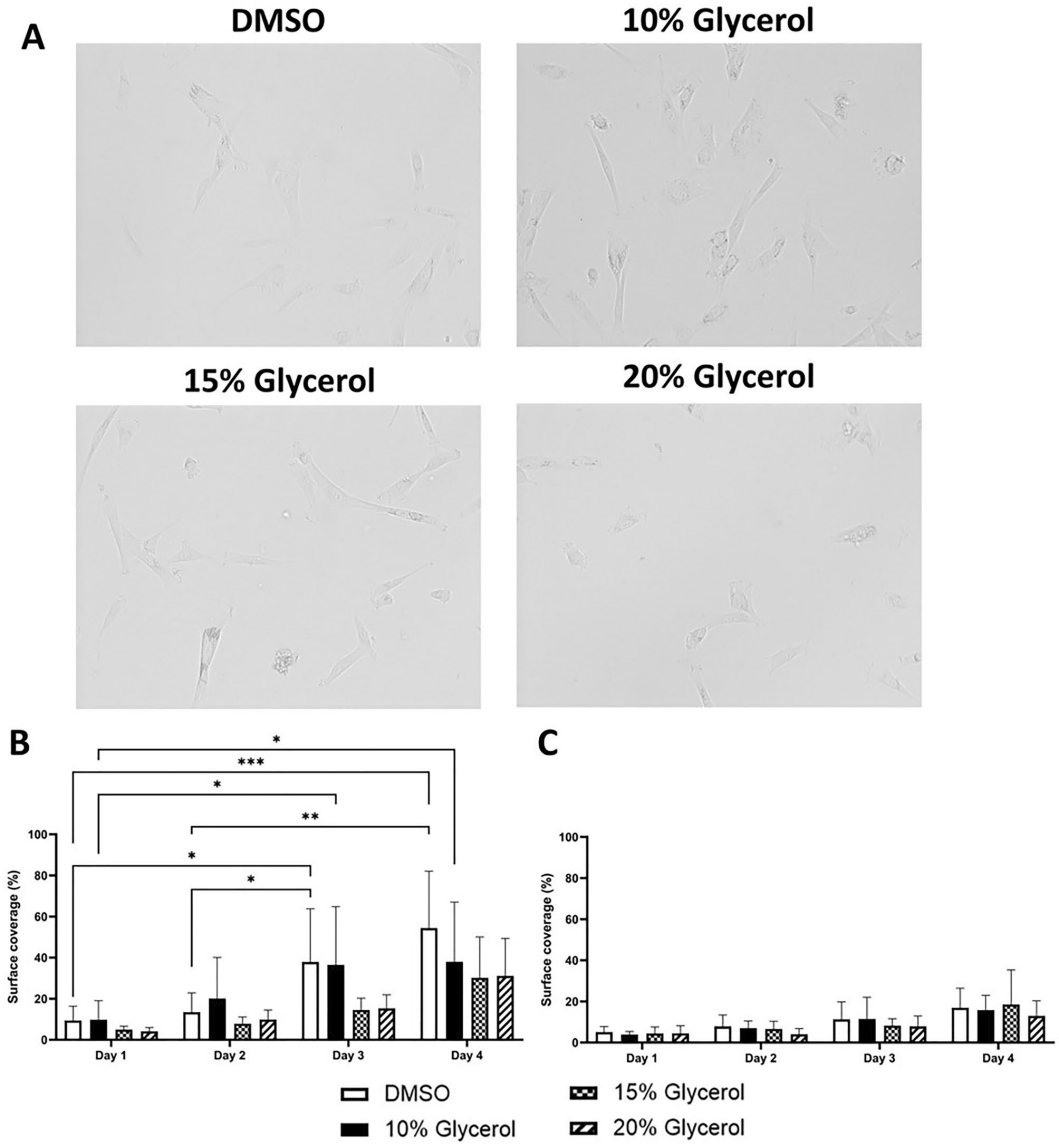
Representative images of the hCS-MSCs morphology and quantified spreading after thawing from being cryopreserved with different CPAs (10% DMSO, 10%, 15% and 20% Glycerol). (**A**) hCS-MSCs morphology, surface coverage in (**B**) complete DMEM, and (**C**) complete MEM media (GMP media). Data expressed as average ± SD of 3 primary hCS-MSCs lines (*p < 0.05, **p < 0.01, ***p < 0.001).

**Figure 5:**
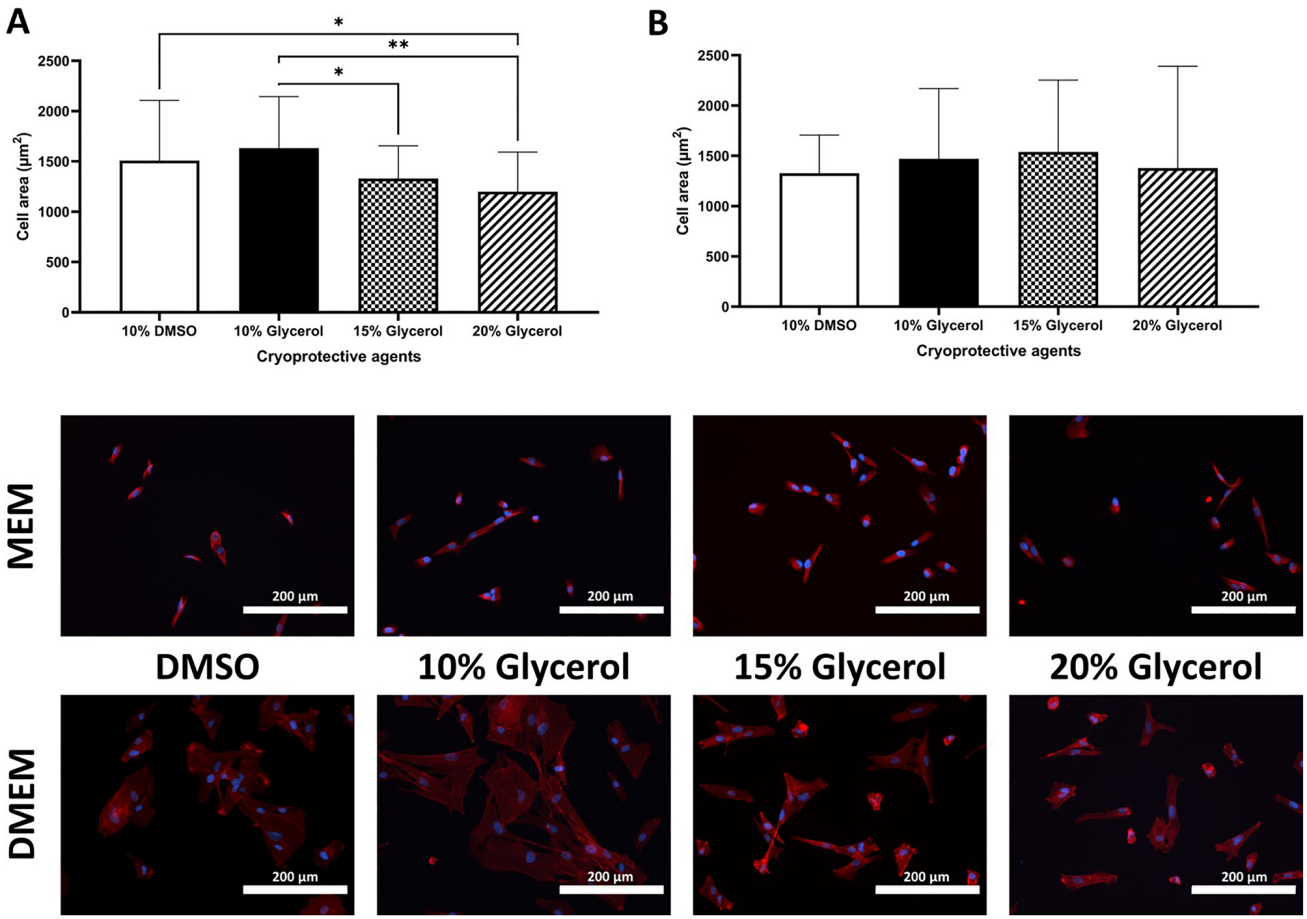
Human CS-MSCs spreading after thawing. The cells were in (**A**) complete DMEM, or (**B**) complete MEM media (GMP media) from cryopreservation in different CPAs (10% DMSO, 10%, 15% and 20% Glycerol), and immunofluorescent images of each condition (Vimentin staining = Red, DAPI nuclear staining = Blue). Scale bar = 200 µm. Data expressed as average ± SD of 3 primary hCS-MSCs lines (*p < 0.05, **p < 0.01).

**Figure 6:**
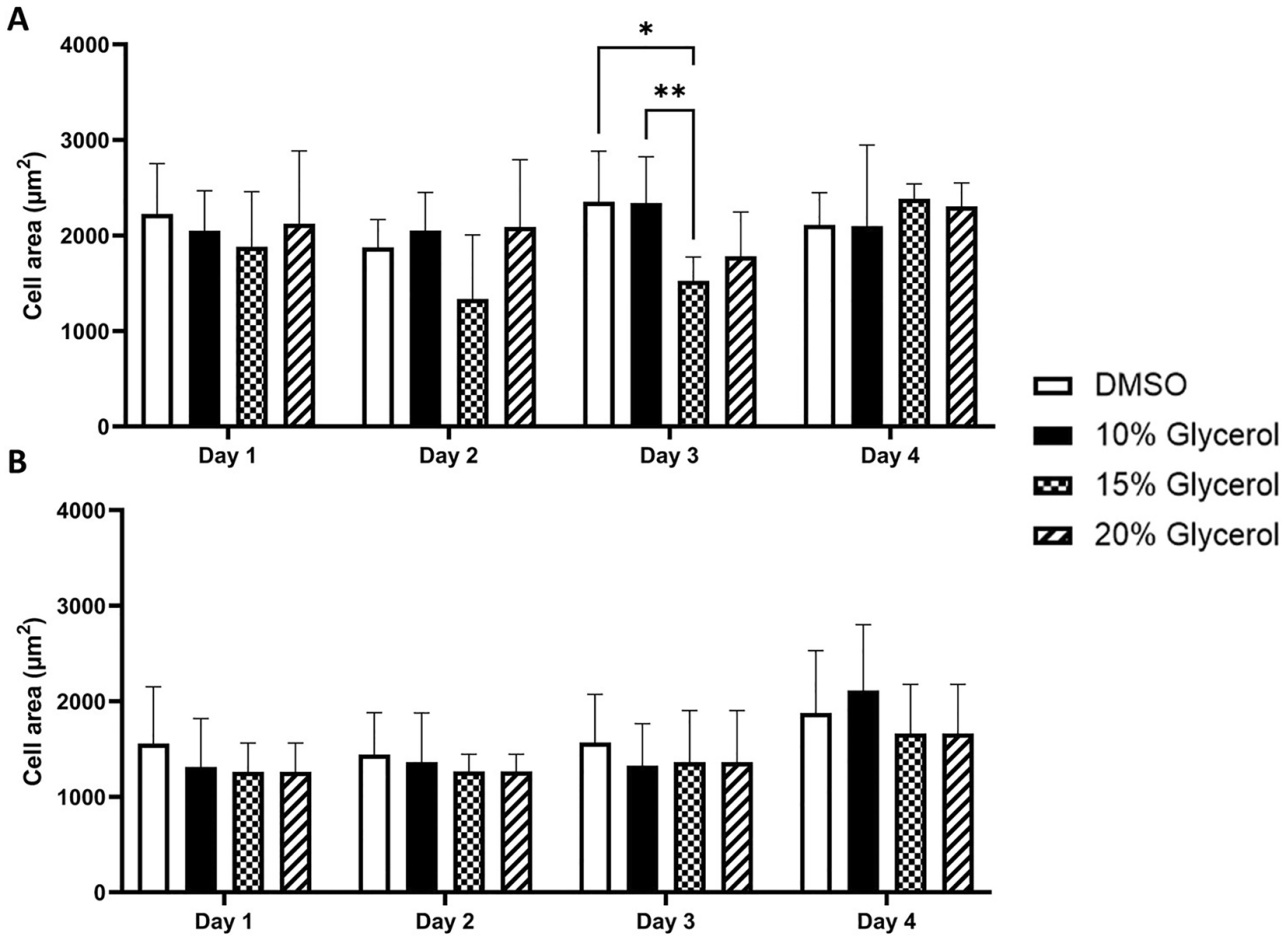
The hCS-MSCs plastic culture surface coverage (%) in T25 flasks after thawing the cells from being cryopreserved using different CPAs (10% DMSO, as well as 10, 15 and 20% Glycerol). (**A**) Complete DMEM media, (**B**) complete MEM media (GMP media). Data expressed as average ± SD of 2- 3 primary hCS-MSCs lines.

The hCS-MSCs plastic culture surface coverage in the T25 flask during the 4 days after thawing the cells cryopreserved in different CPAs and media (complete MEM or DMEM) showed that the cells were able to grow and cover the surface in the flask in an increasing manner over the observation period (Figs. 4, 5, 6).

The hCS-MSCs cryopreserved with any CPA in complete DMEM showed 20–60% of surface coverage at Day 3 and 4 (Fig. 4B), while the cells cryopreserved with any CPA in complete MEM showed values around 20% on Day 4 (Fig. 4C). The hCS-MSCs in complete DMEM were able to cover a larger surface area than those in complete MEM within the first 4 days after thawing (Fig. 4). The hCS-MSCs cryopreserved with DMSO and 10% glycerol in DMEM had a significant increase in surface coverage from Day 1 to Day 4 (Fig. 4B).

The immunofluorescent stainings and the brightfield images presented here on different days of growth after thawing the hCS-MSCs, showed that the cells have a fibroblastoid morphology (Figs. 4 and 5). Analysis of the immunofluorescent stainings of the hCS-MSCs at the day 0 after seeding them cryopreserved with any CPA in complete DMEM or MEM showed cells with a cellular area of approximately 1500 µm^2^ (Fig. 5). In addition, the cells cryopreserved with 10% DMSO in complete DMEM proved to be significantly bigger than those cryopreserved with 20% glycerol, while the hCS-MSCs cryopreserved with 10% glycerol were significantly more spread than the cells cryopreserved in 15% and 20% glycerol (Fig. 5A). Furthermore, the hCS-MSCs cryopreserved in complete DMEM or MEM supplemented with 10% glycerol were able to differentiate towards chondrogenic, adipogenic and osteogenic lineages (See Supplementary Fig. S2).

The data obtained with the brightfield images during the 4 days of experiments showed similar results (Fig. 6). The hCS-MSCs preserved with any CPA in complete DMEM demonstrated to have cell surface area values of approx. 2000 µm^2^ from Day 1 to 4 (Fig. 6A), and the hCS-MSCs cryopreserved with 10% DMSO or 10% glycerol in complete DMEM showed a significantly higher cell surface area coverage than hCS-MSCs cryopreserved with 15% glycerol in complete DMEM at Day 3 (Fig. 6A). Remarkably, the hCS-MSCs cell surface area with any of the CPAs in complete DMEM at any time-point showed to be approximately 500 µm^2^ higher than hCS-MSCs with any CPA in complete MEM at any time point (Fig. 6). In complete MEM, the hCS-MSCs preserved with any CPA appeared to have a cell surface area coverage of approximately 1500 µm^2^ at any time-point (Fig. 6B).

## Discussion

It is known that the use of CPAs for long-term storage of cells is important to preserve the immunophenotypical characteristics of the cells, their viability and ability to proliferate^26^. Cryopreservation of cells is an important stress factor that can affect the permeabilization of their cell membrane^26,27^. Nowadays, there are many alternative CPAs and methodologies to preserve cells, and it is crucial to adjust the CPA methodologies according to the cell type that is being used^20^. In the present study, glycerol was used as an alternative CPA to preserve hCS-MSCs, and it was compared to the gold standard (DMSO).

The International Society for Cellular Therapy has proposed minimal criteria for defining MSCs^28,29^. These parameters are based on plastic culture plate adherence and ≥ 95% of the MSC population expressing CD73, CD90 and CD105, while ≤ 2% should express CD34, CD45, CD11b or CD14, CD79 or CD19 and HLA class II^28,29^. Likewise, the multipotent differentiation of the cells needs to be demonstrated by immunostaining in vitro^28,29^. The immunophenotype characterization of hCS-MSCs cryopreserved with 10% DMSO or 10% glycerol in complete DMEM and complete MEM showed that hCS-MSCs possess the main characteristics of MSCs, which have been described previously by us and others^7,30^. However, it was possible to observe certain differences between the cells preserved with 10% DMSO and 10% glycerol in complete MEM media, and 10% glycerol in complete DMEM media when compared to the values obtained by Vereb et al.^7^. The hCS-MSCs cryopreserved with both 10% DMSO and 10% glycerol in complete MEM media expressed CD146 values closer to the ones found in the literature^7^. Additionally, the presence of CD117 (an early progenitor marker) increased on hCS-MSCs cryopreserved with 10% glycerol in complete MEM, reaching similar values to those found in the literature^7^. The hCS-MSCs cryopreserved with 10% DMSO and 10% glycerol in complete MEM media showed a significant increase in CD184/CXCR4 reaching values of 16% and 97%, respectively. This finding somewhat differs from the data presented by the researchers who described these cells for the first time^7^. Nevertheless, CD184/CXCR4 is known to disappear during cell isolation and subculturing, and some authors have demonstrated a later recovery of this chemokine receptor^31^. CD184/CXCR4 is an important marker involved in many cellular conditions and types of cells, and it is expressed in stem cells and cancer cells^32,33^. This marker has also been shown to have an important role in tissue regeneration (e.g. nervous tissue, heart, lung and liver)^34^, and it mediates progenitor cell homing and recruitment to injury sites, being involved in cell arrest, survival and angiogenesis^35^. Therefore, the indicated effects of CD184/CXCR4 and the ligand CXCL12 can be considered valuable when discovering the characteristics of hCS-MSCs cryopreserved in complete MEM media and more significantly, glycerol. The presented results indicate that glycerol as a CPA could be a better cryoprotectant than the gold standard (DMSO) for hCS-MSCs, the latter previously being described as the best cryoprotectant by many authors^26,36–42^.

The survival rate of the hCS-MSCs cryopreserved with the different CPAs in complete DMEM or complete MEM showed no statistical differences with an approximate loss of 20% of the viable cells, in line with the described loss of viable cells after cryopreservation in the literature, depending on the cell type and the CPA used^43^. Several authors have found a higher loss of cells (30% viable cells)^44^, while others have shown a survival rate of approximately 80–90%^45,46^. It is known that the survival rate may vary depending on the cryopreservation method being used^47^.

The cell adhesion ability of the cryopreserved hCS-MSCs in the different CPAs in complete DMEM or MEM showed that the cells preserved with 10% glycerol have better adhesion qualities than those frozen with 10% DMSO. Other have shown similar findings, observing that MSCs have better attachment qualities after preserving them in alternative CPAs other than DMSO^48^. This supports the hypothesis that 10% glycerol could be a better CPA than DMSO for hCS-MSCs.

There were no differences in the proliferation of the hCS-MSCs with any of the CPAs in complete DMEM, but in line with the previous findings, hCS-MSCs preserved with 10% glycerol in complete MEM demonstrated a significantly better proliferation compared to those cryopreserved with DMSO. Human platelet lysate (HPL) can replace FBS for culturing human MSCs showing better capabilities such as increased cell proliferation, differentiation potential, without affecting the MSC immunophenotype, and potential of immunomodulation among other important factors^12,49,50^. The use of HPL, in addition, helps to reach GMP grade in cryopreservation^12,51^.

The fibroblastoid characteristics of the hCS-MSCs are in line with the fact that these cells can migrate to a damaged area^52,53^. When hCS-MSCs are intact in the cornea, the cells present a dendritic morphology and low proliferation rate^54,55^. When the cornea suffers an injury, the hCS-MSCs attain a fibroblastic morphology, analogous to the hCS-MSCs being cultured in vitro^52,53^.

The hCS-MSCs stored with 20% glycerol presented a spheroid morphology more often than the other glycerol concentrations and DMSO. Although unclear why, it is likely the higher concentration of glycerol could be a key factor contributing to such a morphology^25^. The high viscosity of the glycerol could promote such differences in morphology as seen with other viscous solutions^56^ among other effects^25^. As hCS-MSCs are stromal cells, one should consider higher cell spreading as a beneficial quality, since stromal cells are involved in maintaining the structural integrity of the cornea and the healing of damaged tissue^57^.

The hCS-MSCs appeared more spread in complete DMEM compared to in complete MEM, and the cells preserved with 10% DMSO and 10% glycerol most extensive spreading among all tested conditions. This fact, in addition to the higher proliferation of hCS-MSCs in presence of HPL in complete MEM, could also suggest that hCS-MSCs have a spheroid morphology due to the rapid growth of the cells. Moreover, a higher proliferation rate, could mean hCS-MSCs are dedicating more energy to their reproduction, copying the DNA and other cellular processes related to cells’ division, rather than employing energy on spreading and attaching to a larger surface^58^.

Altogether, the present study shows that we could successfully seek and test an alternative CPA for hCS-MSCs in complete DMEM or MEM. The preservation of hCS-MSCs with 10% glycerol in complete DMEM or MEM demonstrated a very similar survival rate, surface coverage and cell area compared to the hCS-MSCs cryopreserved by 10% DMSO in complete MEM or DMEM. The hCS-MSCs cryopreserved in complete DMEM could cover a larger cell surface area than cells in complete MEMThis can be beneficial in terms of the function of any stromal cells, as well as their spreading and generation of extracellular matrix. The larger size of the cells could be a consequence of their slower reproduction, and further research is required to better understand why the cells present such a morphology in complete DMEM instead of complete MEM. MSCs have a variable growth depending on the composition of the media and the presence of human-derived cell media components instead of components derived from animals^12,51^. Despite these facts, hCS-MSCs cryopreserved with 10% glycerol in complete DMEM or MEM presented similar capabilities compared to those stored with the gold standard DMSO. Additionally, hCS-MSCs cryopreserved by 10% glycerol in complete MEM demonstrated a better cellular adhesion, and in some cases better cell proliferation and recovery of the migratory marker (CD184/CXCR4) compared to cells cryopreserved with 10% DMSO. The cryopreservation with 10% glycerol, independent of the media chosen, demonstrated to be a non-toxic CPA alternative that could be utilized to preserve hCS-MSCs, and eventually be used to store these cells for future cell-therapy following proper GMP guidelines.

Other cells types need to be tested and validated under the same cryopreserving conditions in separate experiments to show general applicability of our findings.

## Methods

### Isolation and cultivation of cells

The hCS-MSCs were isolated from cadaver eyes obtained as leftover material from Norwegian Eye Bank (following DSAEK operation). The processing and use of the human tissue are in accordance with the directives of the Helsinki Declaration and all tissue harvesting was approved by the Regional Committee for Medical and Health Research Ethics (REK 2017/418) in Norway. The hCS-MSCs isolation was performed following our own methodology described by Nagymihaly et al.^59^. Briefly, the corneal disc was separated from the corneal scleral ring, placed in a Petri dish with Dulbecco’s Phosphate Buffered Saline (DPBS) 1X, and the epithelial and Descemet’s membranes were removed by scratching under a stereomicroscope, rinsed and cut in approximately 10–12 pieces. The tissue pieces were then transferred to a microplate of 6 wells (Corning^®^, Axygen^®^, Merck/Sigma-Aldrich, MO, USA) placing 1 or 2 pieces in each well with 800 µL of Dulbecco’s modified eagle medium (DMEM) low glucose with Glutamax™ supplemented with 10% (v/v) fetal bovine serum (FBS; Gibco^®^, Thermo Fisher Scientific, MA, USA) and 1% antibiotic/antimycotic solution (Merck/Sigma-Aldrich, MO, USA) (complete DMEM) and incubated at 37 °C 5% CO_2_ for 24 h. After the first day in culture, the wells were carefully filled up with 2–3 mL of pre-warmed complete DMEM, and the culture media was changed every 3–4 days. The adherent hCS-MSCs from the attached tissue pieces were trypsinized, filtered with a strainer with at least 70 µm pore size, and cryopreserved in liquid nitrogen at -196 °C until further use, approximately 25–30 days after the isolation day. The isolated hCS-MSCs were phenotypically characterized by flow cytometry using a Becton Dickinson (BD) FACS Canto II (BD biosciences, USA) and BD Stemflow Human MSC Analysis Kit (BD biosciences, USA) prior to the experiments.

The hCS-MSCs were cultured in complete DMEM or in Minimum Essential Medium (MEMα, Gibco^®^, Thermo Fisher Scientific, MA, USA) supplemented with 5% (pooled) human platelet lysate (HPL) (Cook Regentec, IN, USA), 1 mM sodium pyruvate (100 mM, 07555 A20, Thermo Fisher Scientific, MA, USA), 2 mM L-Glutamine (200 mM, Merck/ Sigma-Aldrich, MO, USA) and 5 μg/mL gentamycin (50 mg/mL, Merck/ Sigma-Aldrich, MO, USA) (complete MEM), and the media was changed every 2–3 days during cell expansion. The cells were then trypsinized by TrypLE Express Enzyme (Gibco^®^, Thermo Fisher Scientific, MA, USA) at approximately 80% confluency to avoid trans-differentiation and losing their MSC phenotype^59^.

### Storage of hCS-MSCs by applying different cryoprotective agents

The hCS-MSCs were frozen using two different CPAs: standard 10% DMSO (Merck/Sigma-Aldrich, MO, USA) in complete DMEM or MEM, and glycerol (Merck/Sigma-Aldrich, MO, USA) at three different concentrations (10, 15, and 20%) in complete DMEM or MEM (see Table 1 for the experimental setup of the media used).

**Table 1:** Different cell culturing media and cryoprotective agents (CPAs) used in the experiments.

Culture media	CPA
Complete MEM	10% DMSO
	10% Glycerol
	15% Glycerol
	20% Glycerol
Complete DMEM	10% DMSO
	10% Glycerol
	15% Glycerol
	20% Glycerol

Three primary hCS-MSCs donors were counted with a Bürker chamber using Tryphan Blue to visualize the live cells and, subsequently, approximately 7.5·10^5^–1·10^6^ cells/mL were cryopreserved in media containing DMSO or glycerol. The hCS-MSCs cryopreserved in DMSO were gently mixed and immediately transferred into − 80 °C inside a freezing container (Mr. Frosty; 1 °C/min cooling rate), while the hCS-MSCs cryopreserved with glycerol were gently mixed in the cryovial and left on the bench for 20 min prior to transferring into − 80 °C inside the Mr. Frosty. All the cryovials were transferred after a few days into liquid nitrogen for at least two weeks storage.

### Cell surface marker phenotype characterization of the hCS-MSCs

The immunophenotype characterization of the hCS-MSCs was carried out using a BD Accuri™ C6 personal flow cytometer (Becton Dickinson, NJ, USA). FITC, PE and APC- conjugated antibodies (Biolegend, CA, USA) were obtained, against cell surface- expressed proteins: CD31, CD34, CD44, CD45, CD73, CD90, CD105, CD106, CD117, CD146, CD166, CD184 (Sup. Table 1).

Briefly, 1·10^5^ dissociated cells were pipetted into individual 1.5 mL tubes (Corning^®^ Axygen^®^, Merck/Sigma-Aldrich, MO, USA), rinsed and pelleted in cold fluorescence-activated cell sorting (FACS) buffer (0.5% bovine serum albumin (Thermo Fisher Scientific, MA, USA) in DPBS (1X)). Antibody solutions (1:40) were added to the individual tubes with the cells, gently resuspended and incubated on ice for 30 min. Once completed, the hCS-MSCs were washed twice in cold FACS buffer, centrifuged (3 min, 500 RCF g, 4 °C) then resuspended in 0.2 mL buffer before measurement. The immunophenotype characterization was performed with all the primary hCS-MSCs cryopreserved with cryopreserved with 10% DMSO and 10% glycerol, in complete DMEM and MEM, respectively. The data are presented as mean ± standard deviation (SD) values.

### Quantification of hCS-MSCs

The hCS-MSCs cryopreserved with the different CPAs were rapidly thawed in a water bath after 2–4 weeks in liquid nitrogen and counted using a Bürker chamber by Trypan blue to calculate the survival rate ((cells alive after thawing/cells alive before freezing) × 100). Subsequently, the live cells were transferred into 48 well plates to study the cell adhesion after 6 h incubation, and the proliferation assay from day 1 to day 4. At the end of each time-point, the hCS-MSCs were rinsed twice with DPBS (1X) and lysed by 300 μL of mammalian-protein extraction reagent (Thermo Fisher Scientific, MA, USA). Once all samples were collected, the relative number of attached cells was quantified by measuring the lactate dehydrogenase (LDH) activity using the Cytotoxicity Detection KitPLUS LDH (Roche Applied Science, Penzberg, Germany). The samples were measured spectrophotometrically at 492 nm using a Victor 3 microplate reader (PerkinElmer, Waltham, MA, USA), and the number of cells were calculated using the calibration curve prepared with known cell numbers. All the experimental conditions were performed in triplicate.

### Human CS-MSCs trilineage differentiation

Human CS-MSCs cryopreserved with 10% glycerol were differentiated to three lineages using adipogenesis, chondrogenesis and osteogenesis kits (Gibco, Stem- Pro, Thermo Fisher Scientific, MA, USA). The kit media were replaced every 3–4 days for a period of 2–3 weeks in a 48-well plate. Once the differentiation period ended, the media were removed and the cells were fixated in 4% formalin solution. Alizarin Red S (Merck/Sigma-Aldrich), Alcian Blue solution (Merck/Sigma-Aldrich), and HCS LipidTOX (Invitrogen, Thermo Fisher Scientific, MA, USA) were used to stain fixated hCS-MSCs for osteogenesis, chondrogenesis, and adipogenesis, respectively. Images were taken using an EVOS FL fluorescent microscope or a Zeiss upright light microscope.

### Cell surface covering, cell spreading and image analysis

In parallel to the cell adhesion and proliferation assays, 50 000 cells were placed in culture in T-25 flasks to quantify the surface coverage at days 1, 2, 3 and 4 through brightfield microscopy images. Moreover, 20 000 cells were seeded in duplicates in a 48 well plate, and after 6 h incubation, the attached hCS-MSCs were rinsed twice with DPBS (1X), fixed with 10% formalin for 10 min, washed 3 times, and the cells were stained with Vimentin (MA5-16,409, Rabbit Monoclonal, 1:200, Thermo Fisher Scientific, MA, USA) and DAPI (D1306, dilution 1:1000, Thermo Fisher Scientific, MA, USA). Images were taken by an EVOS FL fluorescent microscope (Thermo Fisher Scientific, MA, USA), and further analyzed using ImageJ 1.51w software (NIH, Bethesda, MD, USA) to determine cell area and surface coverage.

### Statistical analysis

All the data are presented as mean values ± SD. ANOVA with multiple comparisons HSD-Tukey test was used to determine statistically significant differences (Graphpad Prism 9, GraphPad Software, Inc., San Diego, CA). Statistical significance was set at p-value < 0.05.

### Supplementary Information

Supplementary Information.

## Data Availability

The original contributions presented in the study are included in the article/Supplementary Material; further inquiries can be directed to the corresponding author.
