# Supplementary material for: Alternative cryoprotective agent for corneal stroma-derived mesenchymal stromal cells for clinical applications
Source: Sci Rep. 2024 Jul 9;14:15788. doi: 10.1038/s41598-024-65469-4 (PMC11233711; doi:10.1038/s41598-024-65469-4)
Supplement: Supplementary file 1 — Supplementary Information. [file 41598_2024_65469_MOESM1_ESM.docx]

**Study of an Alternative Cryoprotective Agent for Corneal Stroma-derived Mesenchymal Stromal Cells for Clinical Applications**

Kristoffer Larsen^1^, Goran Petrovski^1,2,3,4^, Gerard Boix-Lemonche^1,*^

^1^ Center for Eye Research and Innovative Diagnostics, Department of Ophthalmology, Institute of Clinical Medicine, Faculty of Medicine, University of Oslo, Oslo, Norway

^2^ Department of Ophthalmology, Oslo University Hospital, Oslo, Norway

^3^ University of Split, School of Medicine, 21000 Split, Croatia

^4^ UKLONetwork, University St. Kliment Ohridski – Bitola, 7000 Bitola, North Macedonia

* Correspondence and requests for materials should be addressed to G.B-L. (email: gerardboixlemonche@gmail.com)

| **Index** | |
| --- | --- |
| Supplementary Table S1 | 2 |
| Supplementary Figure S1 | 3 |
| Supplementary Figure S2 | 4 |

## Supplementary Table S1

| ***Supplementary Table S1:*** *Surface protein immunophenotype markers studied on hCS-MSCs according to the literature* (7)*.* | |
| --- | --- |
| **Immunophenotype marker** | **Description of the marker** |
| CD31 | Endothelial |
| CD34 | Hematopoietic lineage |
| CD44 | Cell adhesion molecule (CAM) |
| CD45 | Hematopoietic lineage |
| CD73 | Mesenchymal stem cell |
| CD90 | Mesenchymal stem cell |
| CD105 | Mesenchymal stem cell |
| CD106 | Vascular cell adhesion molecule 1 |
| CD117 | Early progenitor marker |
| CD146 | Melanoma/ pericyte |
| CD166 | CAM |
| CD184 | Cell migration |

## Supplementary Figure S1

| 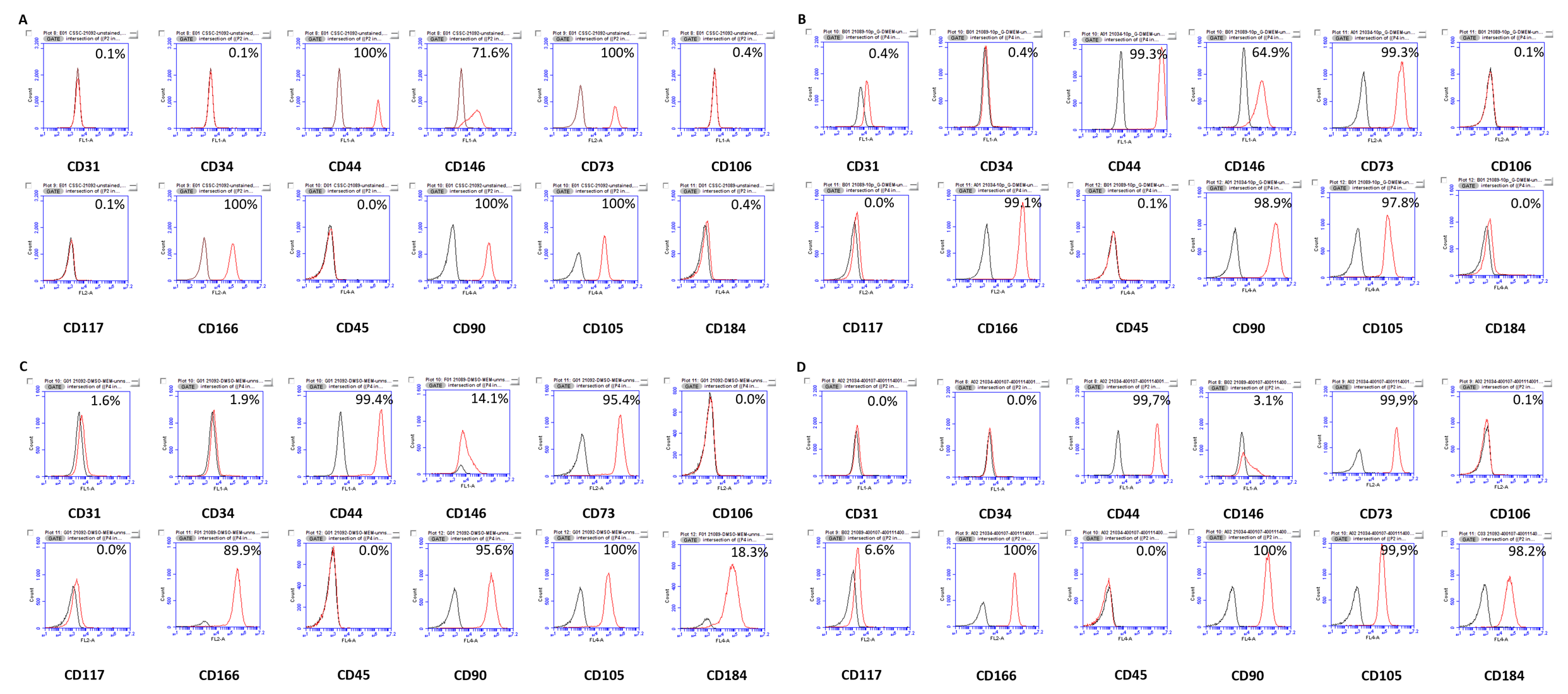 |
| --- |
| ***Supplementary Figure S1.*** *Individual donors’ surface protein immunophenotype profile of cells. The isotype control is visualized by the black line and the signal measures from the hCS-MSCs are visualized as a red line. Figures were exported from CFlow Plus software. (****A****) hCS-MSCs cryopreserved with 10 % DMSO, and (****B****) hCS-MSCs cryopreseved with 10 % glycerol in complete DMEM and (****C****) hCS-MSCs cryopreserved with 10% DMSO, and (****D)*** *hCS-MSCs cryopreserved with 10% glycerol in complete MEM.* |

## Supplementary Figure S2


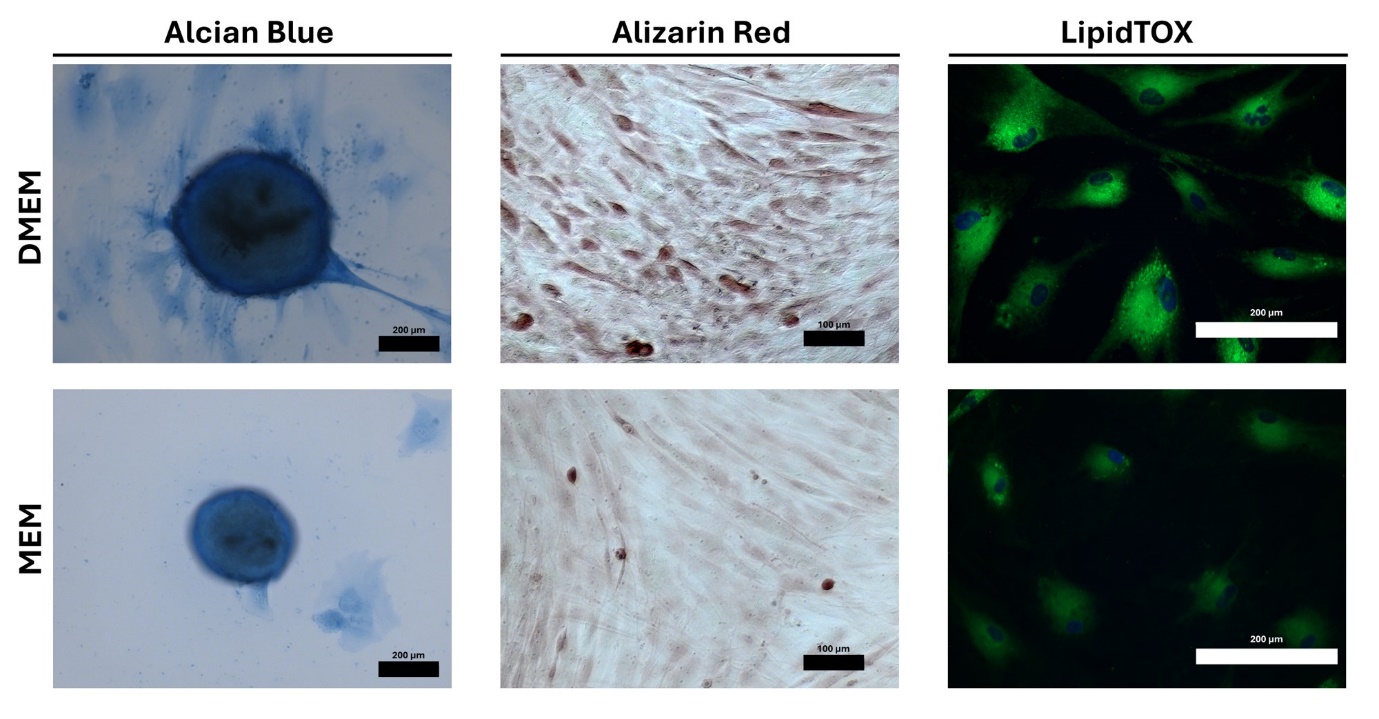


***Supplementary Figure S2.*** *Human CS-MSCs trilineage differentiate. Representative images of hCS-MSCs cryopreserved in complete DMEM and MEM supplemented with 10% glycerol, after induction of differentiation to adipogenic lineage stained by LipidTOX dye and DAPI (intracellular lipids in green, cell nuclei in blue), photographed by an EVOS FL microscope at 20x magnification (white bars = 200 µm); to osteogenic differentiation, visualized by Alizarin Red staining; and to chondrogenic induction, stained with Alcian at 20X magnifications, using a Zeiss microscope.*
